# Experimentally induced metamorphosis in axolotls reduces regenerative rate and fidelity

**DOI:** 10.1002/reg2.8

**Published:** 2014-02-20

**Authors:** James R. Monaghan, Adrian C. Stier, François Michonneau, Matthew D. Smith, Bret Pasch, Malcolm Maden, Ashley W. Seifert

**Affiliations:** ^1^ Department of BiologyUniversity of Florida223 Bartram Hall, P.O. Box 118525GainesvilleFlorida32610USA; ^2^ Nexus Biology GroupUniversity of Florida223 Bartram Hall, P.O. Box 118525GainesvilleFlorida32610USA; ^3^ Florida Museum of Natural HistoryUniversity of FloridaGainesvilleFlorida32611USA

**Keywords:** Axolotl, cell cycle, limb, metamorphosis, regeneration, salamander

## Abstract

While most tetrapods are unable to regenerate severed body parts, amphibians display a remarkable ability to regenerate an array of structures. Frogs can regenerate appendages as larva, but they lose this ability around metamorphosis. In contrast, salamanders regenerate appendages as larva, juveniles, and adults. However, the extent to which fundamental traits (e.g., metamorphosis, body size, aging, etc.) restrict regenerative ability remains contentious. Here we utilize the ability of normally paedomorphic adult axolotls (*Ambystoma mexicanum*) to undergo induced metamorphosis by thyroxine exposure to test how metamorphosis and body size affects regeneration in age‐matched paedomorphic and metamorphic individuals. We show that body size does not affect regeneration in adult axolotls, but metamorphosis causes a twofold reduction in regeneration rate, and lead to carpal and digit malformations. Furthermore, we find evidence that metamorphic blastemal cells may take longer to traverse the cell cycle and display a lower proliferative rate. This study identifies the axolotl as a powerful system to study how metamorphosis restricts regeneration independently of developmental stage, body size, and age; and more broadly how metamorphosis affects tissue‐specific changes.

## Introduction

The ability of some animals to regenerate missing body parts has fascinated scientists for centuries, and the clinical value of tissue regeneration has driven this topic to the forefront of current scientific inquiry (Davenport 2005). Appendage regeneration is widespread among metazoans, and the ability to regenerate damaged tissue was probably present in basal vertebrates (Bely and Nyberg 2010). However, appendage regeneration is deficient or absent in most extant vertebrates. This begs the question: what factors constrain regeneration in some animals? Although a great deal of research has been aimed at understanding the cellular and molecular mechanisms of regeneration, few studies have addressed how fundamental organismal traits such as body size, age, or stage of the life‐cycle may affect regenerative ability. Interpreting how these factors influence regeneration in some species could reveal the key cellular and molecular mechanisms that could promote regeneration in other animals.

The limb has long served as a classic model to elucidate the cellular mechanisms that control appendage regeneration (Rose 1944; Polezhayev 1946; Wallace 1981; Stocum and Cameron 2011). Following limb amputation, epithelial cells migrate from the cut edge and cover the wound surface; this forms the wound epithelium, which is a specialized signaling center required for successful regeneration. Beneath the wound epithelium, cells are stimulated to re‐enter the cell cycle (Globus et al. 1980) and accumulate to form a blastema, a mass of lineage‐restricted progenitor cells that will eventually replace the missing limb (reviewed in Monaghan and Maden 2012). As blastemal cells proliferate they integrate patterning and growth signals, eventually differentiating to form a fully patterned miniature limb that will continue growing. Examining species where limb regeneration has been lost or restricted suggests that cellular changes during ontogenetic development have curtailed the ability to mount a regenerative response and replace damaged tissue (Goss 1987; Sánchez Alvarado 2000; Bely 2010; Seifert et al. 2012a; Seifert and Voss 2013).

Supporting this idea, studies of appendage regeneration across numerous animal models have documented a correlation between ontogenetic development and progressive loss of regenerative ability. Embryonic and larval anamniotes (fishes and amphibians) exhibit an almost limitless ability to regenerate their appendages, and mammalian embryos are capable of regenerating digit tips (Han et al. 2003). In contrast, while some fishes, lizards and urodeles are capable of regenerating appendages as adults, frogs and mammals lose the ability either just prior to metamorphosis (frogs) (Dent 1962) or during post embryonic development (mammals) (Han et al. 2008). Interestingly, metamorphosis imposes similar constraints on regeneration among the distantly related arthropods (insects, arachnids, and crustaceans) (Maruzzo et al. 2005) suggesting that metamorphic transformation alters the cellular response to injury. How exactly these ontogenetic shifts affect the regulatory control of regeneration is poorly understood.

Metamorphosis represents the postembryonic transformation of tissues and physiological systems as larva abruptly transition to juveniles. In amphibians, these spectacular changes are stimulated by thyroxine which binds thyroid hormone receptors attached to DNA and activates latent developmental programs in target tissues (Buchholz et al. 2006). Studies in frogs and salamanders have produced two seemingly opposing viewpoints regarding the effect of metamorphosis on regenerative ability in these groups (Wallace 1981). On the one hand, studies in frogs provide a clear picture that progressive loss of regenerative ability in the limb is a consequence of morphological, cellular, and genomic changes that occur during metamorphosis. First, there is a stage dependent loss of regenerative ability approaching metamorphic climax (Forsyth 1946; Dent 1962; Muneoka et al. 1986). Second, the larval skin matures to a terrestrial form, striated muscle forms, and the skeleton begins to differentiate. As these changes occur, there is a correlated proximal to distal loss of regenerative capacity (Rose 1944; Forsyth 1946; Korneluk and Liversage 1984; Wolfe et al. 2000). Lastly, there is some evidence that metamorphic transformation inhibits gene transcription required for regeneration either directly or through alteration in the epigenetic landscape (Christen and Slack 1997; Yokoyama et al. 2000; Christen et al. 2003; Yakushiji et al. 2007). Thus, developmental changes during metamorphosis drastically reduce regenerative ability in anurans.

In contrast, the prevailing viewpoint for urodeles is that regenerative ability is essentially independent of metamorphosis, as some urodele species can regenerate as adults. Indeed, examination of four species of adult ambystomids found that, under the proper environmental conditions, complete limb regeneration was possible, albeit at a slower rate compared with larval forms (Young et al. 1983a,b). Similarly, most experiments performed with aquatic phase *Notopthalamus viridescens* report perfect regeneration (Wallace 1981). On the other hand, there are contradictory reports of incomplete and abnormal regeneration in species of *Triturus*, *Salamandra*, and *Pleurodeles* (Scadding 1977, 1981; Wallace 1981), and repeated amputations in *N. viridescens* produce skeletal anomalies (Dearlove and Dresden 1976). Moreover, when limb regeneration was assessed in *Pleurodeles waltl* before, during, and after metamorphosis, the result was a clear reduction in regenerative capability as measured by both an increase in digit loss and reduction in the rate of regeneration (Wallace 1981). Whilst these discrepancies may have a phylogenetic explanation, they might also result from age and body size differences in post‐metamorphic animals (Seifert et al. 2012a).

While much has been learned from tadpoles and pre‐ and post‐metamorphic urodeles, each system presents several fundamental limitations that hinder inferences about the direct influence of metamorphosis. First, in tadpoles, when regenerative ability is lost in a stage‐specific manner (i.e., approaching or during metamorphosis) it precisely correlates with the degree of cellular differentiation in the developing limb bud (Muneoka et al. 1986). In fact, some investigators have termed the regenerative response in *Xenopus* and other frogs *embryonic regulation*. This term is used because at regeneration competent stages resident cells have not yet undergone terminal differentiation and are still undergoing embryonic development (Scadding 1977; Korneluk and Liversage 1984). Thus, comparisons between pre‐ and post‐metamorphic anurans inescapably compare embryonic and postembryonic development.

Second, body size and limb size have been implicated as constraints on the rate (and possibly ability) of regeneration (Scott 1909; Pritchett and Dent 1972; Maden 1976; Scadding 1981; Seifert et al. 2012a). In both anurans and urodeles, body size and limb size rapidly increase following metamorphosis; thus comparisons between pre‐ and post‐metamorphic animals are inherently confounded by size effects. Lastly, as it relates to studies in older urodeles, aging itself (either cellular or physiological) may affect regeneration rate and ability, although this relationship is at present poorly understood (Lund et al. 2009; Anchelin et al. 2011; Eguchi et al. 2011; Nachtrab et al. 2011; Itou et al. 2012; Suetsugu‐Maki et al. 2012; Seifert and Voss 2013). With the interaction of so many factors, disentangling the effects of any one property with respect to regenerative ability becomes difficult. To our knowledge, no study has experimentally decoupled embryonic development, body size, aging, and metamorphosis in order to explicitly examine the effects of fundamental traits on appendage regeneration.

Axolotls provide an ideal system to test how body size and metamorphosis affect regeneration. Unlike all anurans and most urodeles, axolotls are a salamander species that exhibit facultative metamorphosis. While axolotls are thought to rarely undergo metamorphosis in the wild, metamorphosis can be induced experimentally (Page and Voss 2009). Furthermore, metamorphosis can be induced during adulthood, allowing one to remove the confounding effects of embryonic development and also to simultaneously control for age‐associated changes in growth, cellular senescence, and cell differentiation. Here, we use this axolotl system to test the hypotheses that body size and thyroxine‐induced metamorphosis lead to (1) a reduction in the rate of regeneration; and (2) a reduction in the ability of the limb to properly undergo regeneration (i.e., successfully progress through defined stages of regeneration and completely replace all missing elements). After finding negative effects of metamorphosis on rate and fidelity, we then tested a third hypothesis to provide a mechanistic explanation for regenerative decline upon metamorphosis: (3) that proliferative rates of blastemal cells are altered in response to thyroxine, such that the ability of blastema cells to replicate and proliferate during regeneration is irreversibly altered.

## Results

### Comparative changes in limb morphology pre and post metamorphosis

**Figure 1:**
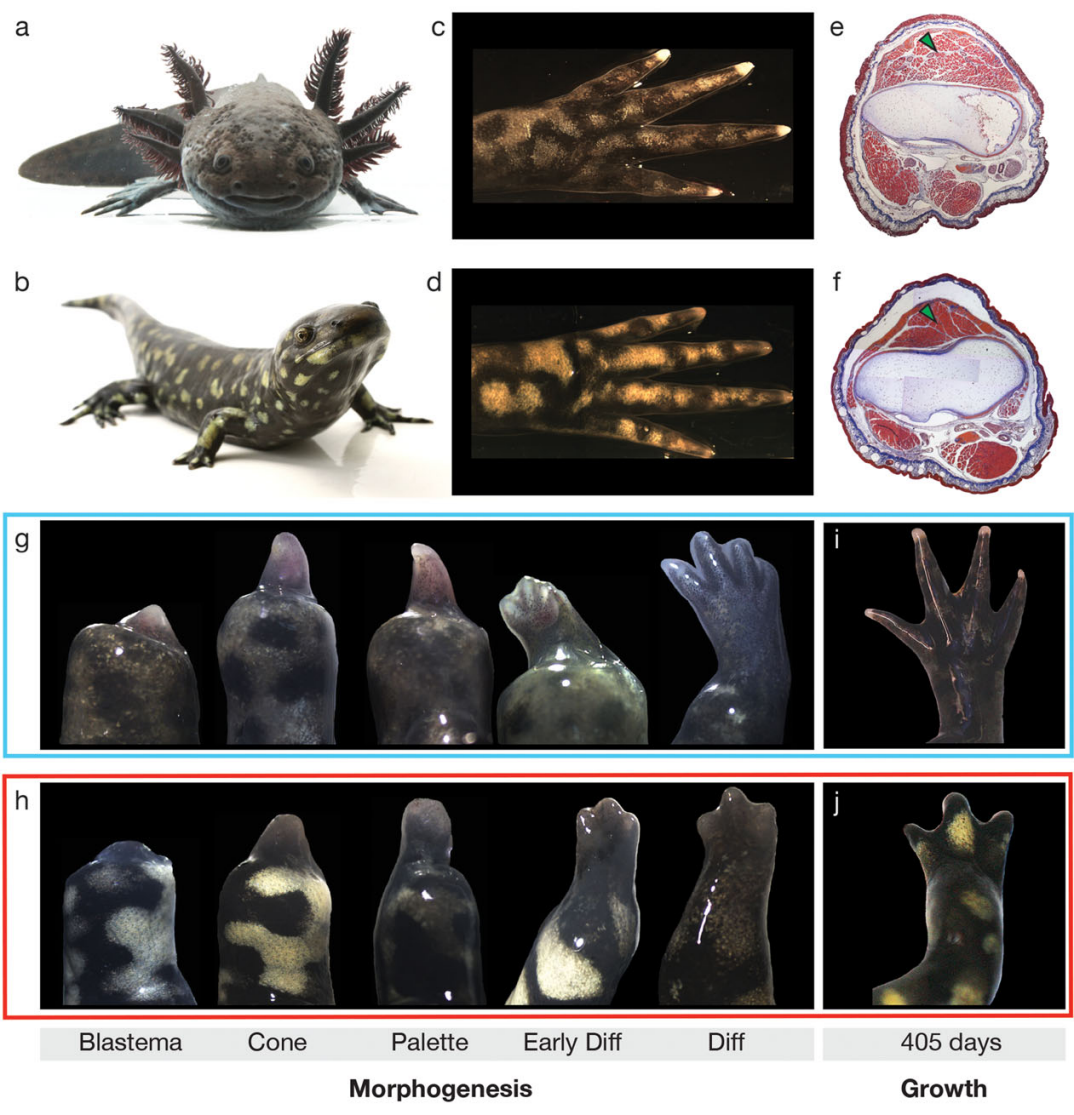
Metamorphosis leads to regenerative defects in axolotls. (A), (B) Representative images of a paedomorph and metamorph at the end of the study. (C), (D) Representative images of a paedomorphic limb (C) and a metamorphic limb (D) at the time of amputation. (E), (F) Histological sections of a paedomorphic limb (E) and metamorphic limb (F) at the amputation plane. Green arrowheads indicate the fasciculi of the anconeus muscle. (G)−(J) Gross morphology of the morphogenesis phase of limb regeneration in paedomorphs (G) and metamorphs (H), and images of the limbs at the end of the experiment (I), (J).

We first identified morphological changes that occur in limbs due to thyroxine‐induced metamorphosis using an age‐matched cohort of 44 young adult axolotls that varied across a broad range of body sizes (Fig. 1A and B). The overlap in body mass (paedomorphs 39−65 g, metamorphs 21−47 g) and snout−vent length (SVL) (paedomorphs 8.5−10.2 cm, metamorphs 8.5−10.6 cm) was similar enough between morphs to allow comparisons of their regenerative abilities. Histological analysis showed some minor differences between the two limb types including: (1) a slightly larger cross‐sectional area in metamorphic limbs [14.2 mm^2^ ± 2.0 standard deviation (SD)] compared with paedomorphic limbs (10.6 mm^2^ ± 1.4 SD; *P* < 0.001) (Fig. S1A); (2) more compaction of the muscle fasciculi in metamorphic limbs (Fig. 1E and F; green arrows); and (3) a lack of Leydig cells in the skin of metamorphic limbs (Jarial 1989; Page et al. 2009; Seifert et al. 2012b). However, we found that pre‐ and post‐metamorphic limb skeletons were both ossified (Fig. 1C−F) and that there was no difference in the proportion of the limb occupied by muscle, skin, and skeletal components near the amputation plane (Fig. S1B and C). In contrast to the drastic changes that occur during metamorphosis in larval anurans, these data suggest that the morphology of paedomorphic and metamorphic axolotl limbs was generally similar prior to amputation.

#### Effects of body size and metamorphosis on regeneration

After an initial acclimation period following metamorphosis, we amputated the right forelimb (just proximal to the elbow) of 44 animals 9 months after hatching. We then examined regeneration rate and ability over a 405‐day period. The analysis of covariance (ANCOVA) testing for the effect of body size (SVL) and metamorphosis on regeneration rate was significant (*F*_3, 37_ = 29.9, *P* < 0.001). The interaction between SVL and metamorphosis was not significant (*P* > 0.14). SVL did not affect the regeneration rate significantly [slope 0.033, standard error (SE) 0.27, *P* = 0.90] while the metamorphic state did (*t* = −9.1, *P* < 0.001). On average, metamorphs reached differentiation at 54.7 ± 25.4 (SE) days, while paedomorphs reached the same stage at 26.2 ± 2.9 (SE) days. When we analyzed each stage separately, we also found no effect of body size on regeneration rate (Table 1).

**Table 1:** Effect of snout−vent length (SVL) and metamorphic treatment on the time between regeneration stages

	Coefficient	Estimate	SE	*t* value	*P* value
Blastema	(Intercept)	13.27	8.57	1.6	0.130
	SVL	0.03	0.09	0.3	0.764
	Metamorphosis	−6.13	0.98	−6.3	**<0.001**
Cone	(Intercept)	18.03	7.74	2.3	0.025
	SVL	−0.11	0.08	−1.3	0.215
	Metamorphosis	−6.13	0.98	−6.3	**<0.001**
Palette	(Intercept)	13.86	7.20	1.9	0.062
	SVL	−0.06	0.08	−0.8	0.443
	Metamorphosis	−3.75	0.84	−4.5	**<0.001**
Early					
differentiation	(Intercept)	35.16	10.70	3.287	0.002
	SVL	−0.32	0.13	−2.7	0.001
	Metamorphosis	−30.70	12.09	−2.5	0.015
	SVL (Metamorphosis	0.303	0.13	2.3	**0.026**
Differentiation	(Intercept)	−3.41	21.49	−0.159	0.875
	SVL	0.25	0.23	1.1	0.294
	Metamorphosis	−12.17	2.45	−5.0	**<0.001**

Values listed are coefficient estimates from an analysis of covariance; where the intercept is listed it represents the estimated time to stage for the paedomorphic limbs. SVL estimates describe the slope of the relationship between SVL and time to stage. Metamorphosis estimates are the change in the intercept for metamorphic limbs. Reported SVL × Metamorphosis coefficient estimates are the estimated difference in slope for metamorphic limbs relative to the slope estimates for paedomorphic limbs. SVL × Metamorphosis coefficient estimates are reported only when significant, because interaction terms were dropped from the model if not significant.

##### Body size does not constrain regenerative rate independent of age

**Figure 2:**
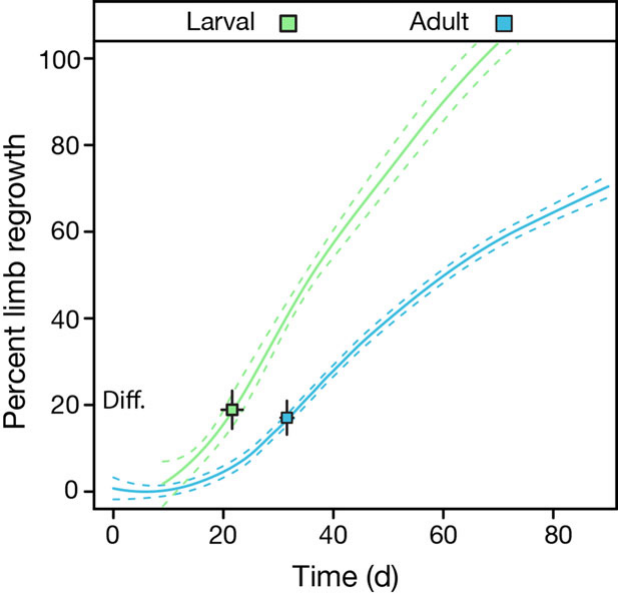
Effect of age on regeneration rate in axolotls. Regeneration rate in larval axolotl regeneration (green line, 3 months post hatching; *n* = 10) compared with older (and larger) adult paedomorphic animals (blue line, 9 months post hatching; *n* = 28). Squares and error bars represent mean ± 95% confidence interval for the percent of the original limb regrown (*y* axis) and the number of days each group took to reach differentiation (Diff.). Colored lines represent a central tendency ± 1 SE from a locally weighted regression to each group (loess, smoothing parameter 0.75).

Our finding that body size had no effect on regeneration rate was at odds with some previous experimental data; thus we performed an auxiliary experiment independently of our initial large‐scale experimental design. We compared regeneration in a group of small larval paedomorphs (3 months post hatching; *n* = 10; average mass 6.2 ± 0.53 SD; average SVL = 51.8 mm ± 4.25 SD) with our larger and older (9 months’ post hatching) paedomorphs (Fig. 2). Larval paedomorphs reached differentiation more quickly at 22.6 ± 3.1 SD days post amputation compared with adult paedomorphs who reached differentiation at 32.0 ± 3.3 SD days (*P* < 0.001 by analysis of variance and Tukey's honest significant difference test). Larval paedomorphs replaced nearly 100% of their amputated limbs 66 days post amputation (average regrowth 98.0% ± 0.1 SD), while adults had regenerated only 56% of their limb over the same time period (Fig. 2; average regrowth 56.0% ± 0.1 SD). This comparison demonstrates that when size is accompanied by an age or developmental stage difference (as has always been the case in previous experiments) size can be interpreted as having a negative effect on regeneration rate.

##### Metamorphosis constrains regenerative rate

**Figure 3:**
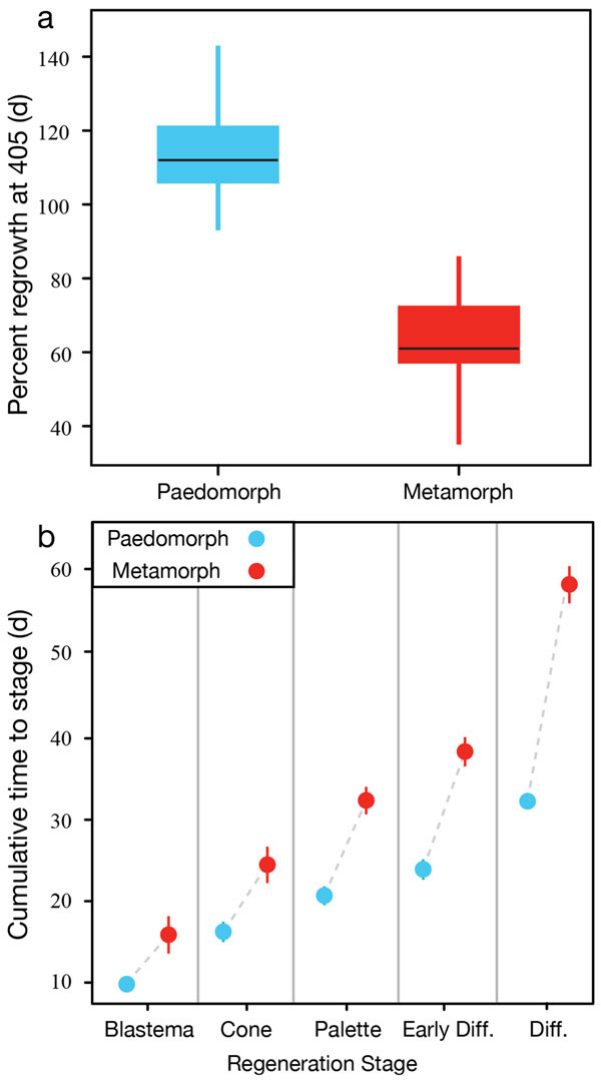
Regeneration rate is reduced following metamorphosis. (A) At 405 days post amputation, paedomorphs regenerated on average 113.6% of the missing limb, while metamorphs regenerated 61.7%. (B) The difference in the cumulative amount of time taken to reach each morphogenesis stage for paedomorphs and metamorphs. Note the increasing difference between morphs through each stage (also see Table 1).

Although limbs of both morphs had passed through all stages of regeneration 405 days after amputation (Fig. 1G−J), we found that metamorphic animals took approximately twice as long to regenerate than paedomorphs (Fig. 3A). During the time paedomorphs took to grow back an entire limb, metamorph regenerates were only 62% of their original size (Figs. 1I, J, and 3A). The failure of metamorphs to completely replace their amputated limb after 405 days was not due to restriction at any one particular stage during regeneration, but rather to an overall slower progression through each stage of regeneration (Fig. 3B). For example, all paedomorphs (*n* = 28) re‐epithelialized the amputation surface within 24 h, while re‐epithelialization in metamorphs (*n* = 15) was not complete until 48−72 h after amputation.

##### Metamorphosis constrains fidelity of regeneration

**Figure 4:**
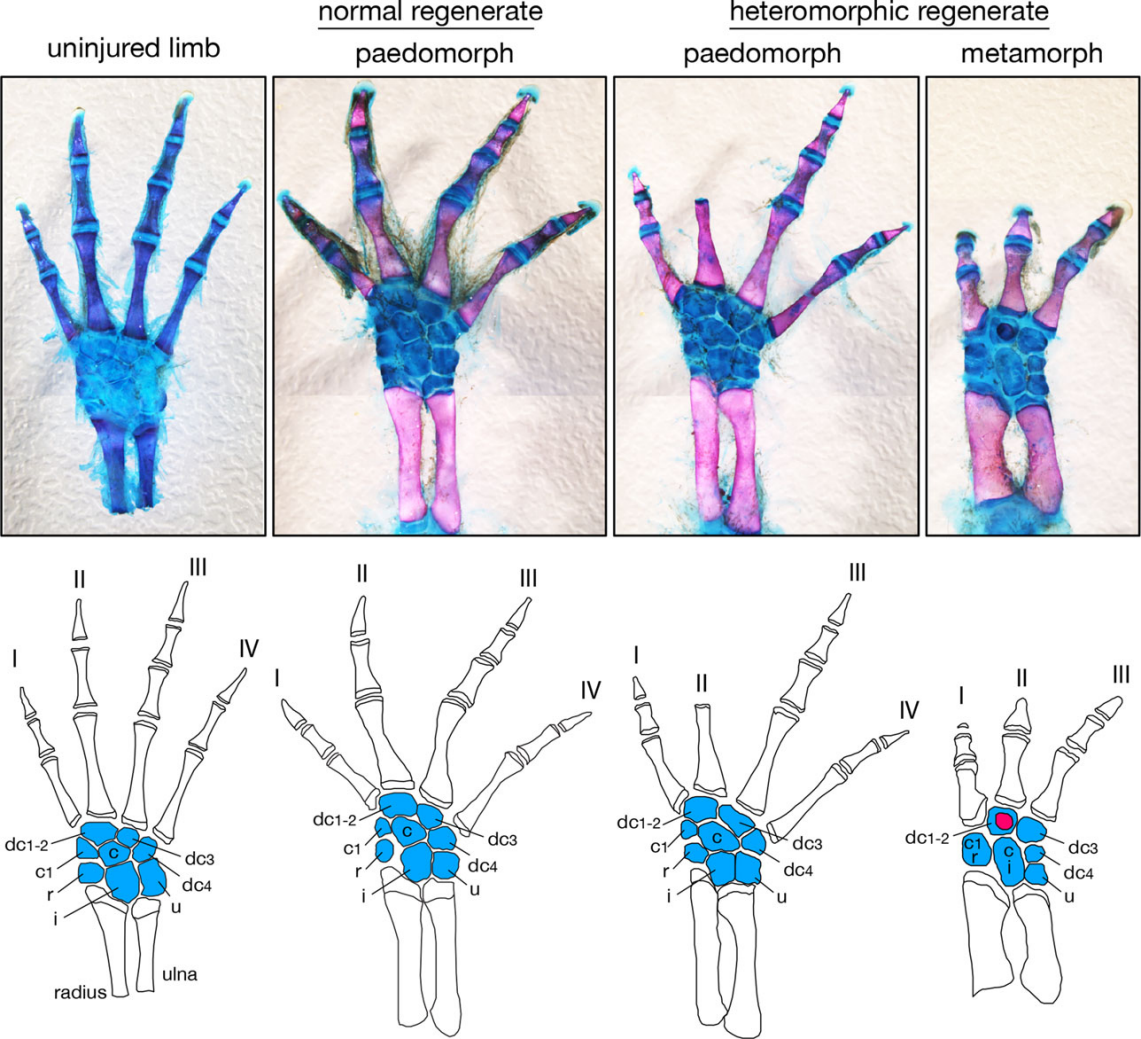
Metamorphosis leads to a reduction in skeletal elements during regeneration. Regenerated right forelimbs viewed dorsally (stained with Alcian Blue and Alizarin Red) 575 days after the original amputation. Skeletal outlines below correspond to the images above. Blue indicates carpals: dc, distal carpal; c1, carpal 1; c, centrale; r, radiale; i, intermedium; u, ulnare. The uninjured metamorph is representative of the normal skeletal pattern for both morphs. The red circle in heteromorphic metamorph is rogue ossification.

Skeletal staining of limbs revealed that the frequency of morphological defects in regenerated limbs was greater for metamorphs compared with paedomorphs (χ^2^_1_ = 11.8, *P* < 0.001). Only 7% of paedomorphs (*n* = 2 of 28) showed slight defects, whereas 100% (*n* = 15) of the metamorphs had moderate to severe anatomical defects (Fig. 4). Ninety‐three percent (*n* = 14 of 15) of metamorphs had a reduction in normal carpal number (Fig. 4). In some cases carpals were completely absent, while in other cases developing carpals failed to separate during morphogenesis such that fusions occurred between the distal carpals and the radiale, ulnare and intermedium. In addition, some individuals developed rogue ossifications within carpals (Fig. 4). Sixty percent (*n* = 9 of 15) of metamorphic individuals were missing digit IV completely, and in two additional cases a truncated digit IV was found growing perpendicularly out of digit III (Fig. S2). In the remaining individuals (*n* = 4 of 15), all four digits were present; however, they were missing multiple phalanges. In contrast to the severity of these defects in metamorphs, the patterning defects observed in paedomorphs were dramatically less severe and were limited to decreased growth of a single digit, resulting in loss of two phalanges on digit II (Fig. 4). These findings demonstrate that thyroxine‐induced metamorphosis disrupts patterning mechanisms necessary for proper limb regeneration.

### Thyroxine‐induced metamorphosis decreases the number of blastema cells in S‐phase

**Figure 5:**
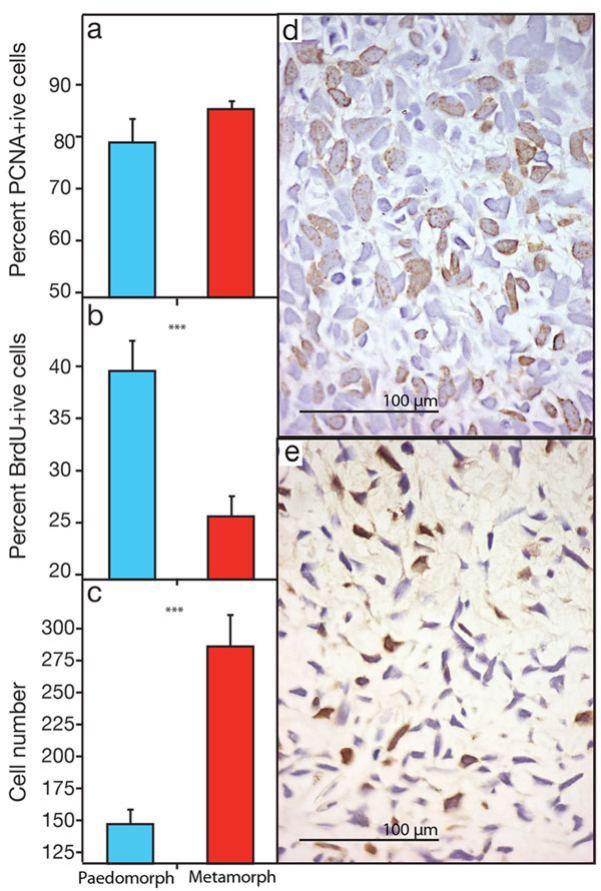
Proliferative rate of blastemal cells is reduced following metamorphosis. Quantification of proliferating cells in a late cone stage blastema. (A) The fraction of total cells that are PNCA positive.  (B) Proliferation rate: the fraction of total cells that are BrdU positive. (C) Total cell density between paedomorphs and metamorphs.  Also shown are histological images of BrdU‐positive cells in (D) metamorphic and (E) paedomorphic blastemas.  Brown nuclei are BrdU positive and blue are hematoxylin‐stained nuclei; scale bar 100 μm.

Given our finding that metamorphosis reduced both regenerative rate and fidelity, we next tested the hypothesis that thyroxine‐induced metamorphosis altered blastema cell proliferation. First, we tested if the total population of proliferating cells (i.e., cells that are in any stage of the cell cycle G1/S/G2/M) differed between morphs by immunolabeling blastema cells with an antibody to proliferating cell nuclear antigen (PCNA). PCNA is a nuclear protein produced during S‐phase that is present throughout all phases of the cell cycle but not expressed in differentiated cells (Barton and Levine 2008). We found that metamorphic and paedomorphic blastemas had a similar fraction of PCNA‐positive cells (Fig. 5A; paedomorphs 78.3% ± 4.5 SE, metamorphs 85.7% ± 1.1 SE; *t* = 1.6, *P* = 0.161), demonstrating that the total proliferating cell population was not different between morphs. Next, we labeled the number of cells that incorporated bromodeoxyuridine (BrdU) in their DNA to test if there were differences in the number of blastema cells in S‐phase. Because total cell cycle length for axolotl blastema cells is approximately 48 h (Maden 1976), we injected animals with BrdU and harvested late cone/early palette regenerates 24 h later to ensure high labeling indices. We counted BrdU‐positive cells in the distal compartment of the blastemas from both morphs (*n* = 6 per group) (Fig. 5B, D, E). We found that metamorphs had a smaller proportion of BrdU‐positive cells (relative to total blastema cells) compared with paedomorphs (paedomorphs 39.8% ± 3.4 SE, metamorphs 24.4% ± 1.9 SE; *t* = 5.0, *P* = 0.001).

Lastly, our finding that fewer metamorphic cells are in S‐phase (BrdU positive) may explain the skeletal defects observed in metamorphs; they could be caused by a reduction in the amount of undifferentiated mesenchymal cells required to replace the missing elements. If this is indeed the case, we would expect to find fewer cells in the metamorph blastema compared with paedomorphs. To test this hypothesis we counted total cell number in the blastema. Contrary to this prediction, we found that metamorphs had a twofold greater number of cells in the blastema (*t* = 5.7; *P* = 0.001).

## Discussion

This study utilized the axolotl as an experimental system to test the effects of thyroxine‐induced metamorphosis on regeneration rate and fidelity. The unique presence of facultative metamorphosis in axolotls allowed us to induce metamorphosis in age‐matched, young adult siblings across a wide range in body size. Although a few previous regeneration studies have found evidence for a negative relationship between body size and regeneration rate (Scott 1909; Pritchett and Dent 1972; Tank et al. 1976; Young et al. 1983b), only one of these studies (that compared forelimbs and hind limbs in the newt) controlled for age (Pritchett and Dent 1972). In contrast to these studies, we found that the range of body sizes studied here does not affect regeneration rate or ability, but our study does not exclude the possibility in other life stages. In fact, our experiment comparing larval animals with young adults showed a clear reduction in regeneration rate. These data implicate age (or developmental stage) as an important modulator of regeneration rate and provide one explanation for why previous studies examining regeneration rate across size classes found a size effect: younger animals, not smaller animals, regenerate at a faster rate.

While we did not find a significant size effect, we found that thyroxine exposure significantly reduced both regeneration rate and fidelity. For metamorphs, we found that although they passed through the typical stages of regeneration, the time necessary to reach any stage from the previous stage was always longer compared with paedomorphs. This suggests that following metamorphosis in urodeles, limb tissue can still mount a regenerative response but the overall rate of morphogenesis and growth is reduced. These results corroborate previous studies in urodeles showing that metamorphic animals regenerate more slowly than larval animals (Goodwin 1946; Seifert et al. 2012b), but here we can pinpoint the observed reduction in rate to the effects of metamorphosis, not because the limb is bigger or that the animal is older. The reduction in regeneration rate that we observed during morphogenesis of the limb (and then subsequently during the growth phase) was probably due to variable growth properties of the limbs between the groups rather than signals coming from outside the limb. Classic experiments that transplanted growing limbs between salamander species have demonstrated that growth of the limb is independent of the overall growth rate for the animal (Harrison 1924; Twitty and Schwind 1931). While the molecular basis for differences in regeneration rate is unknown, one study has correlated expression levels of fibroblast growth factors with variable regeneration rates between urodeles, suggesting a loss of growth‐promoting cues in the regenerating limb (Giampaoli et al. 2003). It is interesting that we observed a correlation between decreased rate and increased morphological defects. Although a link between growth and morphogenesis is possible, we cannot rule out other factors that may affect rate and morphogenesis independently including cellular growth, differentiation, or lack of patterning gene expression. Future studies comparing growth rates between paedomorphic and metamorphic limbs will yield important insight into the control of growth regulation during regeneration and may reveal a link between growth, tissue differentiation, and patterning.

Skeletal analysis of regenerated limbs produced a stark contrast in regenerative fidelity between morphs. One hundred percent of the metamorphs exhibited carpal and digit anomalies. When a digit was lost during regeneration, it was always digit IV, the last digit to form during development (Alberch and Gale 1983). These results show that metamorphosis in axolotls does not prevent a regenerative response, but rather that it negatively affects some component of growth and/or patterning during morphogenesis. With few exceptions (see notes in Towle 1901), most previous studies conducted on a variety of post‐metamorphic urodele species support some type of regenerative response to injury (Goodwin 1946; Dearlove and Dresden 1976; Young 1977; Scadding 1981; Wallace 1981; Young et al. 1983a,b). However, while most (if not all) adult urodele species examined initiate regeneration, there appears to be high variability in the ability to properly complete the process. For example, some families (e.g., Salamandridae, Plethodontidae) appear to regenerate perfectly as post‐metamorphic adults, whereas others (e.g., Ambystomidae, Proteidae, Sirenidae, Amphiumidae) display skeletal anomalies or little regeneration (Towle 1901; Scadding 1977; Young 1977). Attributing this pattern solely to phylogenetic history is tempting, yet previous research also highlights how environmental factors contribute to this variation. For instance, although Scadding (1981) reported no regeneration in several urodele species, Young et al. (1983a) pointed out that improper housing conditions probably inhibited regeneration in some of those species. In addition, Scadding's brief observation period (60 days) was probably too short for regeneration to occur and may have led to the erroneous report of no regeneration in Proteidae or Sirenidae. Lastly, a series of studies looking at four species of Ambystomidae reported normal regeneration in these terrestrial forms. This last case appears to contradict our findings of patterning defects in the closely related axolotl (*Ambystoma mexicanum*); however, close examination of their data suggests that the possibility of reduced carpal or phalange number after dissection was not assessed (Young et al. 1983b). Altogether, there appears to be at least some restriction in the fidelity of regeneration in post‐metamorphic adult urodeles, although the extent of the limitation awaits further testing across a broader phylogenetic range.

In contrast to urodeles, anurans rarely initiate limb regeneration as post‐metamorphic adults, with the exceptions of *Xenopus* (Dent 1962) that regenerate a muscle‐less cartilaginous spike and *Hyperolius* that display digit regeneration (Richards et al. 1975). It is possible that developmental stage, not necessarily metamorphosis, is what limits regenerative ability in these animals. Studies in *Xenopus* and *Rana* have clearly demonstrated a stage‐specific loss of regenerative ability and this loss may be attributed to changes in the mesenchyme of the developing limb (Rose 1944; Agarwal and Niazi 1980; Korneluk and Liversage 1984; Muneoka et al. 1986; Wolfe et al. 2000). For example, while *Xenopus* regenerative mesenchyme (from developing limbs) can induce the expression of genes necessary for limb regeneration in non‐regenerative epithelium, regenerative epithelium cannot induce expression in differentiated mesenchyme (Yokoyama et al. 2000). Furthermore, developmental genes known to promote larval limb regeneration cannot rescue regeneration in post‐metamorphic anuran limbs (Barker and Beck 2009; Slack et al. 2004) unless transgenic overexpression of developmental genes is accompanied by a graft of embryonic limb progenitor cells (Lin et al. 2013). These studies support findings in many vertebrate models that regenerative capacity is lost during development (Seifert and Voss, 2013) and suggest that changes related to cellular differentiation partly underlie loss of regenerative capacity.

Our finding that the number of blastemal cells in S‐phase was reduced in metamorphs suggests that thyroxine‐induced metamorphosis alters how cells function once regeneration has been initiated. Although our analysis was not precise enough to calculate the exact length of the cell cycle, our analysis using PCNA and BrdU allows us to draw some relative inferences. Because the number of blastema cells within any phase of the cell cycle (e.g., PCNA+ cells) was approximately the same between morphs while fewer metamorph cells were in (or entered) S‐phase over a 24‐h period (BrdU+ cells), this suggests that there may be a difference in cell cycle length, probably by an increased G1 transit time (i.e., fewer cells entering S‐phase) and/or a slower progression through G2/M. However, because double‐staining of cells with PCNA and BrdU was not performed we cannot definitively conclude that cell cycle length was different between morphs, and future studies are needed to more precisely address this point. Interestingly, developmental studies in limbs (Ohsugi et al. 1997; Ten Berge et al. 2008; Towers et al. 2008; Zhu et al. 2008), genitalia (Seifert et al. 2010), and brains (Komada et al. 2008) have all documented how disruption of cell cycle dynamics can influence patterning. The fact that metamorphs exhibited patterning defects extends these findings to regeneration.

If thyroxine exposure and subsequent metamorphosis permanently alters cell cycle length, then pharmacological disruption of the cell cycle in paedomorphs should result in patterning defects. Alberch and Gale (1983) inhibited cell division during axolotl hindlimb development using colchicine, a reversible mitotic inhibitor, and observed alteration in skeletal morphology. While variable, they found individual phalanges missing in 90% of the treated limbs and carpal reductions in 32%. In addition, they found posterior digit V was lost in 36% of the treated limbs. Urodele limbs display preaxial dominance during development with the most posterior digit (digit IV in forelimbs and digit V in hindlimbs) specified last. It is interesting that disrupting the cell cycle during development and a lower proliferative rate in post‐metamorphic axolotls always affected the last skeletal elements to form (in addition to other elements in some animals). In addition, Ohsugi et al. (1997) demonstrated that slowing cell cycling in the posterior region of chick limbs also led to patterning defects. These studies, together with our findings, suggest two possible mechanisms for the observed patterning defects. The first mechanism is that there is a minimal mass of cells required to execute proper patterning of the limb, and a reduction in proliferative rate decreases this number below that threshold (Alberch and Gale 1983). The available data are not consistent with this hypothesis. Previous work experimentally manipulating the size of axolotl blastemas during regeneration, and thus reducing the total cell mass, still produce normally patterned limbs (Maden 1981). Additionally, our calculation of total cell number shows the opposite of what is expected under this mechanism: there is a higher cell density in metamorph blastemas compared with paedomorphic blastemas. The second mechanism is that alterations to cell cycle timing directly affect transcription of patterning genes (Ohsugi et al. 1997). While we do not present experimental evidence in support of this second mechanism, comparative work in lizards has shown that decreasing *sonic hedgehog* expression in developing limbs correlates to posterior digit loss and decreased proliferation (Shapiro et al. 2003). Although the relationship between cell cycle regulation and expression of patterning genes is poorly understood, the axolotl system presented here clearly can serve as a tool to explore the complex relationship between patterning and growth during regeneration.

Translating discoveries in regeneration biology into tractable advances in regenerative medicine will probably stem from understanding how regeneration is regulated in animal models where regeneration naturally occurs. Overall, this study demonstrates that fundamental traits of an organism such as metamorphosis and age or developmental stage have important influences on the regenerative capacity of animals. Our findings show that manipulating metamorphosis in the axolotl is a powerful tool for exploring how thyroxine‐induced metamorphosis alters regeneration at a cellular level. Our discovery that metamorphosis produces cellular changes that could be altering cell cycle dynamics and reduces regenerative fidelity warrants further study to identify the genetic or epigenetic control regulating the regenerative response. The system also provides an opportunity to investigate if cellular changes are reversible and if regenerative potential of appendages can be unlocked in mammals.

## Conclusions

Body size and limb size do not affect regeneration rate or ability in adult axolotls.

Adult axolotls regenerate more slowly after undergoing metamorphosis. Our study pinpoints the reduction in rate to the effects of metamorphosis, not because the limb is bigger or that the animal is older.

In conjunction with the above finding, our observation that larval axolotls regenerate faster than young adults supports the contention that developmental stage exerts a strong effect on regeneration rate but does not affect ability in salamanders.

Although regeneration is stimulated, changes associated with metamorphosis reduce the fidelity of regeneration: metamorphs show an inability to correctly execute patterning and growth during regeneration.

Our finding that cell cycle dynamics are different in metamorph blastemal cells compared with paedomorphic cells suggests that metamorphosis alters how axolotl cells function once regeneration has been initiated. Whether these alterations are genetic or epigenetic awaits further testing.

## Materials and Methods

### Animal housing, thyroxine‐induced metamorphosis, and surgeries

Axolotls were obtained from the Ambystoma Genetic Stock Center (University of Kentucky, Lexington, KY) and raised at the University of Florida in accordance with guidelines through the University of Florida Institutional Animal Care and Use Committee. Animals were housed in 40% Holtfreter's solution and maintained on a 12:12 light−dark cycle at a constant temperature (20−22°C). Larval and juvenile axolotls were housed in Tupperware containers and fed live California blackworms *ad libitum* (Lumbriculus). At approximately 5 cm SVL, animals were then moved to Z‐Hab Duo Aquatic Habitats automated flow through systems (Pentair, Apopka, FL) systems and transitioned to feeding on salmon pellets once a day (Rangen, ID).

At 6 months post hatching, thyroxine (50 nmol/L T_4_) was added to the rearing water of axolotls to induce metamorphosis (Sigma, St. Louis, MO) (Page and Voss 2009). Animals were transferred to individual shallow tanks of water through the duration of metamorphosis and monitored daily for metamorphic progress. Once animals finished metamorphosis, they were housed on peat moss and transitioned to moist paper towels in Tupperware containers. Metamorphs were manually fed an entire earthworm twice a week.

Surgeries were performed when animals were either 9 months post hatching (adults and metamorphs) or 3 months post hatching (larval). Paedomorphic axolotls were anesthetized in 0.01% benzocaine (Sigma, St. Louis, MO) and metamorphs anesthetized in 0.02% benzocaine. Surgical scissors were used to amputate the right forelimb just proximal to the elbow and the humerus was trimmed to make the amputation surface flush.

### Morphometrics and data analysis

The amputated limb was imaged at the time of amputation, and the regeneration stump was imaged 24, 48, and 72 h later. Thereafter, it was imaged every third day for 8 weeks, and then bi‐monthly until 405 days. Imaging was done using a Nikon SMz‐U (Nikon, Melville, NY) stereomicroscope equipped with a Leica DFC310 FX digital camera (Leica, Buffalo Grove, IL). Using these digital images, regeneration stage was scored visually through consensus of six observers. The regenerating limb was measured by tracing the outline of the regenerate using an Intuos_4_ pen tablet (Wacom, Vancouver, WA) and processed using ImageJ.

We scored regenerative ability by observing limb regeneration through previously defined stages (Tank et al. 1976; Wallace 1981) that were subdivided into two main phases: a morphogenesis phase and a growth phase (Fig. 1G−J). The morphogenesis phase consists of distinct stages: wound closure (re‐epithelialization); de‐differentiation and accumulation of progenitor cells (blastema formation); proliferation of progenitor cells (cone stage); organization of progenitor cells into precursor tissues, during which patterning can be externally visualized (palette, early differentiation stages); and finally, the generation of a complete skeleton, similar to the missing limb (differentiation stage). The growth phase is characterized by elevated growth of the limb compared with the rest of the body until the miniature limb expands to replace the missing structure (Tank et al. 1976).

To test the effect of body size and metamorphosis on regenerative rate, we performed an ANCOVA on the numbers of days it took individuals to reach differentiation as the response variable, SVL as the covariate and the metamorphic state as the independent variable. SVL was comparable between the two groups (median, metamorphs 90.5 mm, paedomorphs 95.0 mm; SD, metamorphs 3.45, paedomorphs 5.33), and the variances between the two groups were homogeneous (Levene's test, *F*(1, 33) = 0.086, *P* > 0.77). To test for the effect of metamorphosis on regenerative ability, we performed a χ^2^ test using Yate's continuity correction on the number of individuals with normal and heteromorphic limbs for the paedomorphs and metamorphs. For this analysis, given the strong effect of metamorphosis on the proportion of heteromorphic individuals, we could not take into account body size in our analyses. All data analysis and visualization were performed in R 3.0.0 (R Development Core Team 2012).

### Skeletal preparation

Following the completion of the experiment, regenerated limbs were amputated (575 days after the original amputation) and fixed overnight in 10% neutral buffered formalin, washed in phosphate‐buffered saline and prepped by removing skin, muscle, tendon, and ligaments. Limbs were stained overnight in 2% Alcian Blue in ethanol and acetic acid, washed in 100% ethanol, rehydrated through an alcohol series, stained for 2 days with 1:50 solution of 0.1% Alizarin Red in 1% KOH, washed in 1% KOH and cleared in glycerol.

### Histology and Immunohistochemistry

Amputated limbs and blastemas were collected at the time of injury and fixed in 10% neutral buffered formalin overnight at 4°C. Tissue was washed and decalcified in 10% ethylenediaminetetraacetic acid for 3 days with daily changes at 4°C. Following decalcification, tissue was rinsed in phosphate‐buffered saline, transferred to 70% ethanol, and prepared for paraffin embedding. Tissue was sectioned at 5 μm. For routine histological staining, Mason's Trichrome (Richard Allen, Thermo Scientific, Hudson, NY) staining was performed according to the manufacturer's protocol.

For proliferation analysis, animals were staged and, when observed to have late cone/early palettes, were subsequently injected with BrdU (Sigma, St. Louis, MO) (concentration 100 mg/g). Regenerating limbs were harvested 24 h later and processed for paraffin embedding as above. Sections were deparaffinized, blocked for endogenous peroxidase activity in 3% H_2_O_2_ in methanol for 10 min, rehydrated, antigen retrieved by heating in pH 6.0 sodium citrate buffer for 25 min, rinsed in water, incubated in 37°C 2 mol/L HCl for 15 min, rinsed thoroughly in water, rinsed with TBS (tris‐buffered saline), blocked with rabbit serum, blocked for endogenous avidin and biotin, incubated with primary antibody rat anti‐BrdU (1:500, Accurate Scientific, Westbury, NY), washed, incubated with biotinylated secondary anti‐rat (1:400, Vector Scientific, Burlingame, CA), washed and visualized using Vector ABC horseradish peroxidase and DAB (3,3'‐diaminobenzidine) reagents according to the manufacturer's instructions. Tissue sections were counterstained with hematoxylin (Vector Scientific, Burlingame, CA). Other tissue sections collected from the same animal were incubated with mouse anti‐PCNA (1:2000, Dako M0879, Carpinteria, CA), washed, incubated with biotinylated secondary anti‐mouse (1:400, Vector Scientific Burlingame, CA), washed and visualized using Vector ABC horseradish peroxidase and DAB reagents.

For calculating proliferative index, three 40× field of view pictures were randomly taken in the distal third of the later cone/palette stage tissue section for each of six biological replicates. BrdU‐positive or PCNA‐positive cells were counted, followed by total cell number. Relative numbers of BrdU‐ or PCNA‐positive cells were calculated as (#BrdU‐ or PCNA‐positive cells/total number of cells) × 100. Cell density was calculated as the total number of cells per counting frame. Both proliferative index and cell density were averaged across the three frames for each individual.

## Supporting information

Disclaimer: Supplementary materials have been peer‐reviewed but not copyedited.

**Figure S1**. Similarities between metamorphic and paedomorphic limbs.

**Figure S2**.  Example of fused digit III/IV in regenerated metamorphic limbs.
