# Supplementary figures and images for: Experimentally induced metamorphosis in axolotls reduces regenerative rate and fidelity
Source: Regeneration (Oxf). 2014 Feb 20;1(1):2–14. doi: 10.1002/reg2.8 (PMC4895291; doi:10.1002/reg2.8)

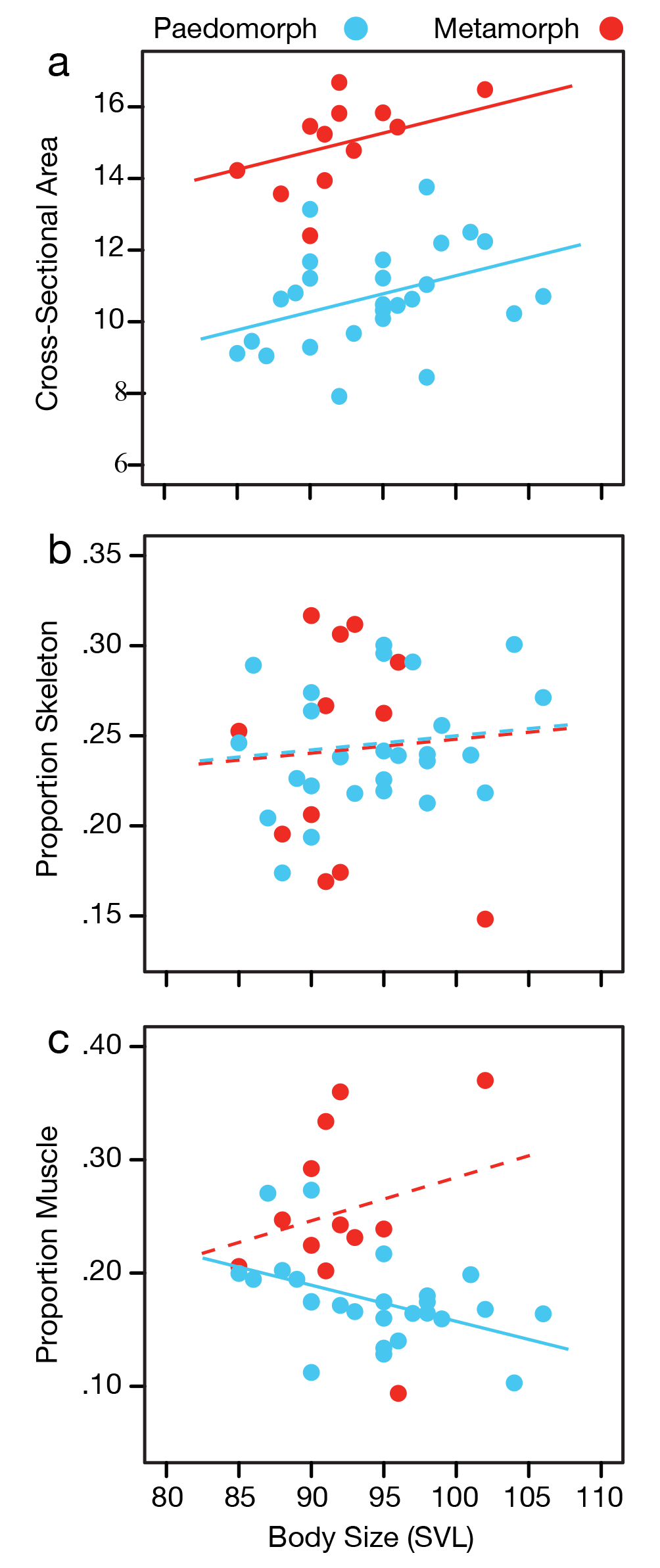

Supplement: Supplementary file 1 — Figure S1. Similarities between metamorphic and paedomorphic limbs. [file REG2-1-02-s001.tif]

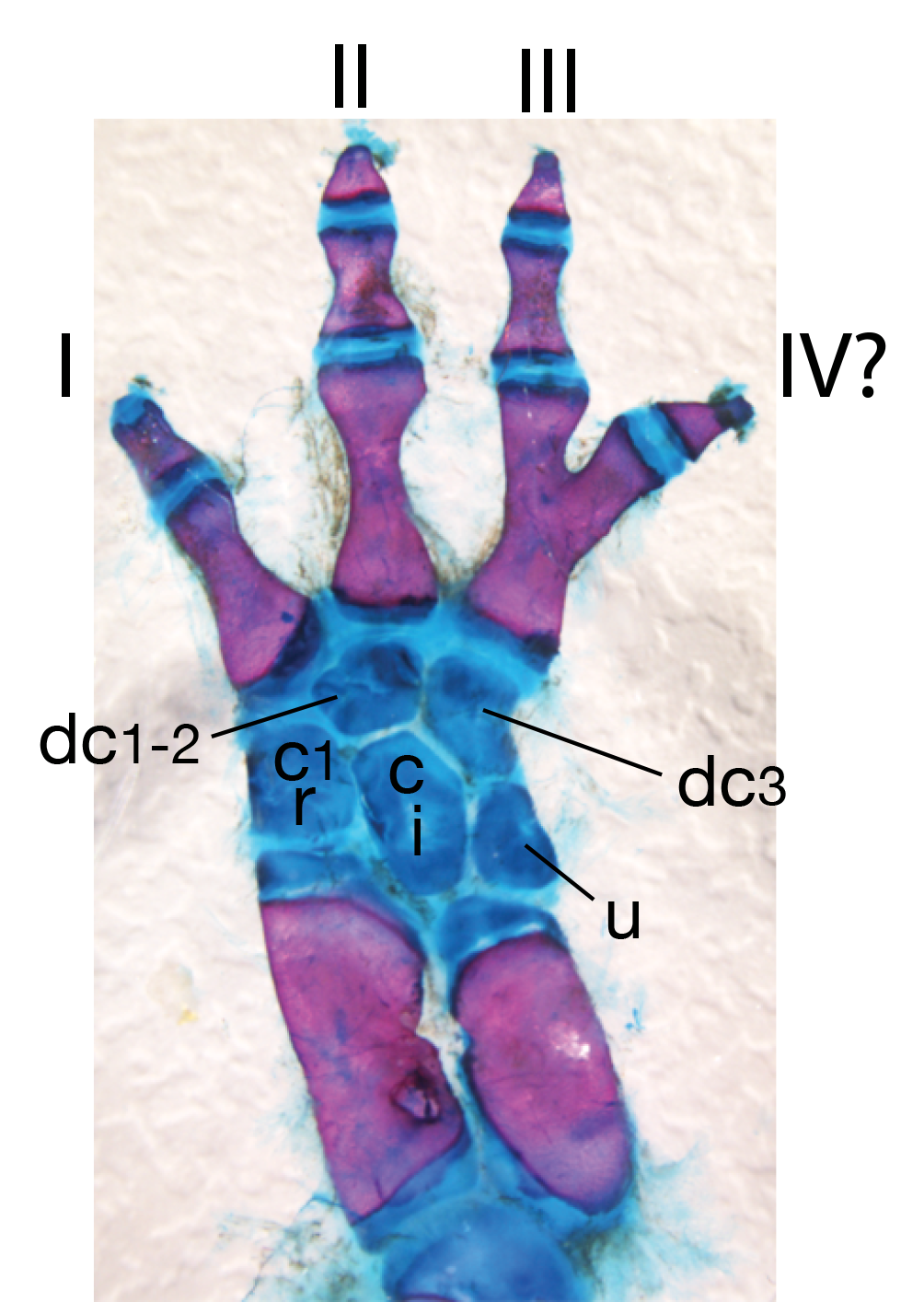

Supplement: Supplementary file 2 — Figure S2. Example of fused digit III/IV in regenerated metamorphic limbs. [file REG2-1-02-s002.tif]
